# *EZH2* alterations in follicular lymphoma: biological and clinical correlations

**DOI:** 10.1038/bcj.2017.32

**Published:** 2017-04-21

**Authors:** S Huet, L Xerri, B Tesson, S Mareschal, S Taix, L Mescam-Mancini, E Sohier, M Carrère, J Lazarovici, O Casasnovas, L Tonon, S Boyault, S Hayette, C Haioun, B Fabiani, A Viari, F Jardin, G Salles

**Affiliations:** 1 Hospices Civils de Lyon, Laboratoire d'Hématologie, Pierre Bénite, France; 2 INSERM1052, CNRS 5286, Université Claude Bernard, Faculté de Médecine Lyon Sud Charles Mérieux, Université de Lyon, Pierre Bénite, France; 3 Department of Bio-Pathology, Hematology, and Tumor Immunology, Institut Paoli-Calmettes and Aix-Marseille University, Marseille, France; 4 Institut Carnot CALYM, Pierre Bénite, France; 5 Department of Hematology, Henri Becquerel Comprehensive Cancer Center and Normandie Univ, UNIROUEN, Inserm U1245, Rouen, France; 6 Synergie Lyon Cancer, Bio-Informatics Platform, Centre Léon Bérard, Lyon, France; 7 Département de Recherche Translationnelle et de l'Innovation, Génomique des Cancers, Centre Léon Bérard, Lyon, France; 8 Département d'Hématologie, Gustave-Roussy, Université Paris-Seclay, Villejuif, France; 9 Department of Hematology and INSERM UMR1231, Hôpital Le Bocage, University Hospital of Dijon, Dijon, France; 10 Lymphoid Malignancies Unit, Hôpital Henri Mondor, University Hospital, APHP, Créteil, France; 11 Department of Pathology, Centre Hospitalier Saint Antoine, Paris, France; 12 Hospices Civils de Lyon, Service d'Hématologie Clinique Pavillon 1 F, Centre Hospitalier Lyon Sud, Pierre Bénite, France

## Abstract

The histone methyltransferase EZH2 has an essential role in the development of follicular lymphoma (FL). Recurrent gain-of-function mutations in *EZH2* have been described in 25% of FL patients and induce aberrant methylation of histone H3 lysine 27 (H3K27). We evaluated the role of *EZH2* genomic gains in FL biology. Using RNA sequencing, Sanger sequencing and SNP-arrays, the mutation status, copy-number and gene-expression profiles of *EZH2* were assessed in a cohort of 159 FL patients from the PRIMA trial. Immunohistochemical (IHC) EZH2 expression (*n*=55) and H3K27 methylation (*n*=63) profiles were also evaluated. In total, 37% of patients (59/159) harbored an alteration in the *EZH2* gene (mutation *n*=46, gain *n*=23). Both types of alterations were associated with highly similar transcriptional changes, with increased proliferation programs. An H3K27me3/me2 IHC score fully distinguished mutated from wild-type samples, showing its applicability as surrogate for *EZH2* mutation analysis. However, this score did not predict the presence of gains at the *EZH2* locus. The presence of an *EZH2* genetic alteration was an independent factor associated with a longer progression-free survival (hazard ratio 0.58, 95% confidence interval 0.36–0.93, *P*=0.025). We propose that the copy-number status of *EZH2* should also be considered when evaluating patient stratification and selecting patients for EZH2 inhibitor-targeted therapies.

## Introduction

Follicular lymphoma (FL) is the second most common type of B-cell non-Hodgkin lymphoma (NHL), comprising ~25% of all new diagnoses.^1^ Although the development of immunochemotherapy has revolutionized the treatment of FL patients, FL is usually considered to be incurable,^2^ and efforts are emphasized toward understanding lymphomagenesis mechanisms. Next-generation sequencing (NGS) studies have drawn increasing attention to frequent mutations in epigenetic regulators in almost all cases of FL.^3, 4^ Moreover, recent advances in the characterization of the mutational landscape of FL have allowed for the establishment of a composite score (m7-FLIPI), which incorporates the mutational status of seven genes, demonstrating that integrating gene mutations into prognostic models could improve risk stratification for FL patients.^5^ These genes include the histone methyltransferase *EZH2* gene, which encodes the catalytic subunit of the polycomb repressive complex 2 (PRC2) that mediates mono-, di- and tri-methylation of histone H3 lysine 27 (H3K27me1, H3K27me2 and H3K27me3), an epigenetic mark associated with transcriptional silencing.^6, 7, 8^ *EZH2* is often constitutively activated in germinal center (GC)-derived NHLs by gain-of-function mutations within the catalytic SET domain, most notably affecting tyrosine 646 (Y646).^3^ In particular, somatic heterozygous mutations have been described in ~25% of FL cases.^9, 10^

Functional studies revealed that the Y646-mutated protein had a higher affinity for H3K27me2 than the wild-type protein, leading to a widespread redistribution of the H3K27me3 marks and inducing complex transcriptional changes at putative PRC2 target genes.^11, 12, 13, 14, 15^ As *EZH2* appears to regulate the GC reaction by sustaining the function of the activation-induced cytidine deaminase (AID) enzyme and preventing terminal differentiation in GC B cells, it was suggested that dysregulation of the GC reaction by constitutively active *EZH2* may promote lymphomagenesis.^16^ The proposed importance of EZH2 as a possible therapeutic target in NHL led to the development of EZH2 inhibitors for therapeutic use.^13, 17, 18^

Patients with *EZH2* gain-of-function mutations have been proposed as ideal EZH2 inhibitor recipients. However, it has recently been suggested that the H3K27 methylation profile may not be fully correlated with *EZH2* mutation status in diffused large B-cell lymphoma.^19, 20^ Thus, some *EZH2* non-mutated patients may also benefit from EZH2 inhibitor therapy. Copy-number variations at the *EZH2* locus (located at the 7q36.1 region) are rarely reported, despite their potential influence on EZH2 expression level and PRC2 methylation ability. We hypothesized that gains of chromosome 7, reported in ~20–30% of FL patients,^21, 22^ might be important for the assessment of EZH2 catalytic activity and help to determine which patients would benefit most from targeted therapy. Thus, we assessed a cohort of 159 FL patients for (i) the mutation status and copy-number variation at the *EZH2* locus, (ii) the effect of these alterations on global gene expression and (iii) the impact of these alterations on patient outcomes.

## Materials and methods

### Study population

Fresh-frozen tumor-biopsy specimens were obtained from 159 FL patients included in the open-label, international, multicenter randomized PRIMA study, which enrolled 1135 patients with untreated high-tumor burden FL (NCT00140582).^23^ Tumor biopsies were obtained at FL diagnosis and stored at the bio-bank Plateforme de Ressources Biologiques, Henri Mondor Hospital, Créteil (bio-bank ID number: BB-0033-00021). Moreover, formalin-fixed paraffin-embedded (FFPE) biopsy specimens were also available for tissue micro-array (TMA) construction for 116 patients of this cohort. All tissues used for this study came from pre-treatment diagnostic biopsies and were confirmed as FL grade 1–3a^1^ by expert hematopathologists. This study was conducted in accordance with the Declaration of Helsinki and approved by the Comité consultatif de protection des personnes se prêtant à la recherche bio-médicale (Hospices Civils de Lyon). All patients signed a consent form for participation in specific biological studies.

### RNA sequencing

RNA was extracted using the miRNeasy Mini kit (Qiagen, Venlo, the Netherlands) according to the manufacturer's instructions. RNA samples that fulfilled the required quality criteria (RIN>7) were further processed by RNA sequencing (*n*=148). RNA sequencing was performed at the IntegraGen Genomics sequencing platform (Evry, France). A 2 × 75 bp paired-end sequencing library was prepared from 2 μg total RNA using the Illumina TruSeq Stranded mRNA Sample Prep kit (Illumina, San Diego, CA, USA). The protocol of poly(A)+RNA purification, fragmentation, first and second strand cDNA synthesis, adapter ligation and DNA enrichment was performed according to the manufacturer's instructions. Paired-end sequencing was performed on the Illumina HiSeq2000 platform, with a mean number of reads per sample of 132 714 297 (range: 101 002 788–188 916 268).

Genome alignment and variant calling were performed using TopHat,^24^ GATK Haplotype Caller^25^ and Picard (http://broadinstitute.github.io/picard/) software programs. Expression data were assessed using the HTseq library.^26^ Count data normalization was performed using the DESeq2 package^27^ and transformed using the rlogTransformation function in R (version 3.2.2, https://www.r-project.org/).

### *EZH2* mutation screening by genomic PCR and Sanger sequencing

DNA was extracted using the High Pure PCR Template Preparation kit (Roche, Basel, Switzerland) according to the manufacturer's instructions. The mutation statuses of *EZH2* exons 16 and 18 were screened in all samples with available DNA (*n*=156). The *EZH2* mutated residues were numbered according to the transcript NM_004456.4 in the NCBI nucleotide database.

The mutation status of *EZH2* exon 16 was assessed by bidirectional Sanger sequencing from a genomic PCR. The forward primer was designed as previously described by Morin *et al.*,^3^ and the reverse primer sequence was specifically designed (sequence of the forward primer: 5′-TCTCAGCAGCTTTCACGTTG-3′ reverse primer: 5′-CACAAACATGCAGAAGTCCAG-3′). PCR reactions were performed in a 50-μl final volume containing 5 μl of genomic DNA (5 ng/μl), 300 nm of each primer (Eurogentec, Seraing, Belgium), 800 nm dNTP, 2 mm MgCl_2_ and 0.75 μl of AmpliTaq Gold DNA Polymerase (Applied Biosystems, Foster City, CA, USA). PCR was performed on a DNA Engine Thermal Cycler (BioRad, Hercules, CA, USA) using the following conditions: 95 °C for 10 min and 30 cycles of 94 °C for 60 s, 60 °C for 60 s and 72 °C for 60 s. Mutations were confirmed by sequencing from two independent PCR amplicons.

The mutation status of *EZH2* exon 18 was determined by HRM analysis using primers described by Bodor *et al.*^10^ (sequence of the forward primer: 5′-AGGCAAACCCTGAAGAACTG -3′ reverse primer: 5′- GGACTGAAAAGGGAGTTCCA -3′). Briefly, PCR reactions were performed in a 20-μl final volume containing 5 μl of genomic DNA (5 ng/μl), 300 nm of each primer (Eurogentec), 1 μl of Resolight dye and 10 μl of LC480 Probe Master Mix (Roche). PCR was performed on a LC480 thermo-cycler (Roche) using the following conditions: 95 °C for 10 minutes and 45 cycles of 94 °C for 15 s, 60 °C for 15 s and 72 °C for 30 s. Mutational status was assessed by gene scanning analysis and was confirmed by Sanger sequencing for 15 negative cases (10% of all samples) and all positive cases, allowing for the discrimination of potential somatic mutations from known polymorphisms.

### Single-nucleotide polymorphism arrays and copy-number variation analysis

Copy-number abnormalities were assessed in the 156 DNA samples using a high-resolution Cytoscan HD single-nucleotide polymorphism array (Affymetrix, Santa Clara, CA, USA). The data were first pre-processed using the Affymetrix apt-chp-to-txt.exe utility to compute summarized probeset values. Signal intensity values (log R ratio and allelic difference) from single-nucleotide polymorphism (SNP) probesets were then extracted and given as input to GAP^28^ to perform joint segmentation and calling of log R ratio and allelic difference. GAP output is an absolute copy-number call that takes into account the inferred ploidy of the tumor and the allelic imbalances. These absolute copy-number values were then transformed into a normalized copy-number deviation value that takes into account the patient-specific ‘neutral' state of each tumor and sex specificity of the X chromosome using the following formula: CNdev=((CN/medianCN) × 2)-NormalState, where medianCN is the ploidy of the tumor calculated as its median over all probesets CN call, and NormalState=2, except for the chromosome X-specific region in males, where NormalState=1. All annotations were based on the hg19 genome build.

### Immunohistochemistry

Sections from FFPE tissue samples were used to build TMAs. During pathological review of each section, areas containing malignant follicles, representative of the entire biopsy sample and avoiding fibrotic portions were marked on the paraffin blocks. Cylinders that were 1 mm in diameter from three different areas were then collected and included in the TMA blocks. After dewaxing and pressure-cooker antigen retrieval, immunostaining was conducted in an automated immunostainer (Dako, Glostrup, Denmark) using a standard avidin–biotin–peroxidase technique. The primary antibodies (EZH2, H3K27me2 and H3K27me3) and staining conditions were used as previously described.^19^ Deparaffinization, rehydration, epitope retrieval and staining were performed as described by Dubois *et al.*^19^ Slides were scored in a blinded fashion by three experienced anatomopathologists (LX, ST, LM). Tumors were scored according to the proportion of tumor cells stained (0–10, with 0 representing negative staining, 1 representing 1–10% positive tumor cells and 10 representing 91–100% positive tumor cells). For each patient, the methylation score was adapted from Dubois *et al.* as follows:


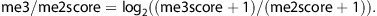


### Statistical analyses

#### Correlation with clinical data and outcome

The correlations between *EZH2* mutational or copy-number status and initial characteristics or treatment group were assessed using Fisher's exact test. Expression levels between groups were compared using the Mann–Whitney test. Correlation between variant allele frequencies (VAF) and H3K27me3/me2 score has been assessed using a Spearman test. Time-to-event parameters (progression-free survival (PFS) and overall survival) were estimated by the Kaplan–Meier product-limit method and compared using a log-rank test. All tests are two-sided.

#### Analysis of over-representation of the expression gene set between groups of patients

Patients were separated into two groups based on alteration status and a transcriptome-wide differential expression was performed using XLSTAT software. We controlled for false positives using a false discovery rate (FDR) threshold of 5%.^29^ Then, the resulting lists of upregulated and downregulated genes in each patient subgroup (carrying a mutation or a gain) were separately assayed to test for enrichment in collections of gene sets from the Molecular Signatures Database v5.2 (MSigDB).^30^ The overlap with MSigDB gene sets was tested using hypergeometric distribution corrected for multiple hypotheses testing with an FDR threshold of 5% as previously described (http://software.broadinstitute.org/gsea/msigdb/annotate.jsp).

## Results

### Clinical characteristics of the *EZH2* study population

The clinical characteristics of the 159 FL patients in this study were comparable to those of the entire PRIMA cohort (1135 patients who received induction treatment), except that the present cohort had less frequent B symptoms (24% in the present cohort versus 32% in the entire PRIMA study, *P*=0.04; Supplementary Table S1). The primary end point (PFS in randomly assigned patients) of the PRIMA study was also evaluated in this cohort. With a median follow-up of 6.6 years from the registration date in the PRIMA trial (inter-quartile range: interval: 6–7 years), the 5-year PFS rate for the present cohort was 67.9% (95% confidence interval (95% CI), 54.4–78.2%) in the rituximab maintenance group and 45.9% (95% CI, 34.9–56.2%) in the observation group (*P*=0.007), in accordance with the results of the entire PRIMA study (Supplementary Figure S1).

### Frequency of *EZH2* genetic alterations

Samples from 148/159 patients were available for RNA sequencing, which yielded a mean coverage of 127 × (range: 15–395 × ) at the *EZH2* locus, with 85% of the bases covered by >15-fold. Mutations were detected in 42 of the 148 patients (28%) in agreement with recent reports.^5, 9, 10^ Of the 42 mutated samples, mutation affected codon Y646 in 38 samples (Y646N, *n*=17; Y646F, *n*=11; Y646S, *n*=6; Y646H, *n*=2; Y646C, *n*=2), codon A692 in 4 samples (A692V, *n*=3; A692L because of double mutation GCA>TTA, *n*=1) and codon W629 (W629G) in one sample also carrying A692V. VAF ranged from 11 to 80%.

We confirmed (in 145 out of the 148 patients with RNA sequencing data) or evaluated (11 patients with no RNA-sequencing data) the mutation status of *EZH2* exons 16 and 18 by genomic PCR in tumor DNA. Of the 44 mutations detected by RNA sequencing, 40 mutations were confirmed at the genomic level, 2 mutations could not be assessed because of the lack of available DNA and 2 mutations were not detected by Sanger sequencing, although the frequency of the mutated transcripts was ~30%. Of the 11 additional patients screened for genomic DNA mutations, a mutation at codon Y646 was detected in 4 patients (Y646S, *n*=2; Y646N, *n*=1; Y646F, *n*=1). Overall, 29% of the patients with available material for one or the other technique (46/159) had a mutation in the *EZH2* gene.

The copy-number status at the *EZH2* locus could be successfully assessed in 155/159 patients (no DNA was available for three patients and one sample was rejected for technical issues). A gain at the *EZH2* locus was detected in 23 (15%) samples as part of the larger gains of chromosome 7 encompassing the 7q36.1 region. No deletion was observed. In total, 37% of patients (59 of 159) harbored an *EZH2* gene alteration—either mutation and/or gain. No association was found between the copy-number and the mutation status. Of note, in the three patients with VAF >50%, two had a gain and one showed a copy-neutral loss of heterozygosity at the *EZH2* locus. Thereafter, the term ‘alteration' is used to indicate the presence of a mutation, a gain or both, whereas ‘no alteration' refers to samples with neither mutation nor gain.

The clinical characteristics at diagnosis of patients with or without an alteration in the *EZH2* gene were comparable (Table 1). However, there was a lower frequency of bone marrow (BM) involvement and B symptoms (BM involvement: 54% versus 70% for patients without alteration, *P*=0.009; B symptoms: 14% versus 30%, *P*=0.02) and a higher frequency of elevated serum lactate dehydrogenase levels (53% versus 28%, *P*=0.04) in patients with an alteration.

### Correlation between *EZH2* genetic alterations, EZH2 protein expression and H3K27 methylation

Using expression data from RNA-seq experiments, we found that the presence of a mutation, a gain or both in the *EZH2* gene was strongly associated with increased expression of *EZH2* mRNA (*P*<0.001 in all cases, Table 2). We then sought to determine the impact of these alterations at the protein level. Owing to the material depletion and stringent interpretation criteria, the total number of patients with immunohistochemistry (IHC) data was lower than expected (74 out of 116 samples), and only 55 and 63 patients samples had interpretable results for EZH2 and H3K27me3/me2 stainings, respectively, with 44 patients with valid data for both assays (Supplementary Figure S2). The protein level of EZH2 assessed by IHC showed a significant increased expression only for patients with a genomic gain (*P*<0.001).

**Figure 1:**
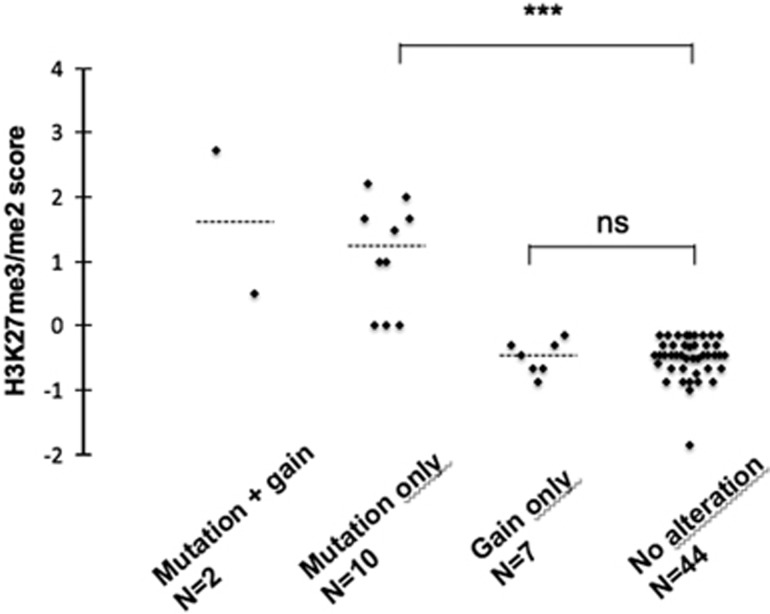
H3K27me3/me2 score according to *EZH2* alteration status. IHC staining of H3K27me3 and H3K27me2 was assessed for 63 patients. Patients carrying a mutation in *EZH2* had a significantly higher H3K27me3/me2 score (median: 1.24 versus −0.46, *P*<0.001, Mann–Whitney test). The dashed line represents the median score in each group.

**Figure 2:**
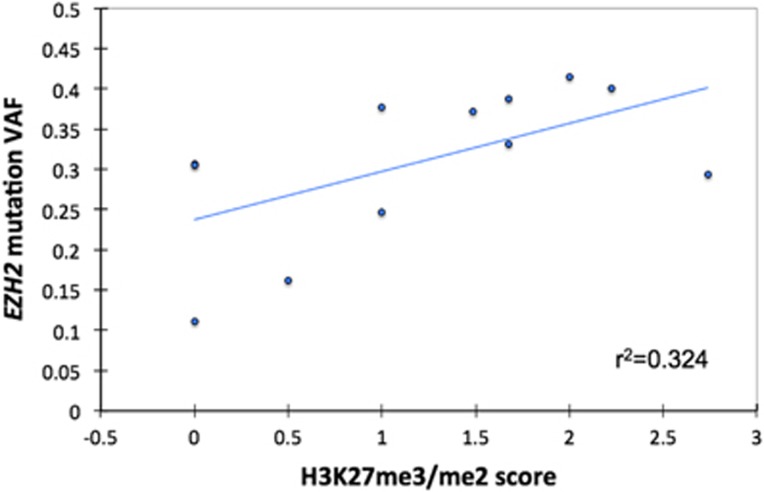
Correlation between the H3K27me3/me2 score and VAF of *EZH2* mutations. The VAF of *EZH2* mutations at the mRNA level showed a slight correlation with the H3K27me3/me2 score assessed by IHC (Spearman-correlation test, *P*=0.056).

The mechanism by which mutated EZH2 protein acts on H3K27 methylation is now well described,^11, 12, 13, 14^ but its precise impact on chromatin structure seems to be more complex than previously proposed.^15^ Given the high prevalence of mutations and genomic gains at the *EZH2* locus, we sought to determine their potential impact on H3K27 methylation. In our cohort, the me3/me2 score range and median were −1.86 to 2.74 and −0.32, respectively (Table 2). The me3/me2 score was significantly higher in mutated samples (samples with mutation-only versus no alteration, *P*<0.001, Figure 1), and it fully distinguished mutated (score ⩾0) samples from wild-type (score <0) samples, with 100% sensitivity and specificity. Interestingly, the VAF, assessed from RNA-seq data, showed a slight correlation with the H3K27me3/me2 score (*P*=0.056, Figure 2). This might reflect either a dose-dependent effect of the mutated EZH2 level on H3K27 methylation, or the proportion of sub-clones carrying an *EZH2* mutation within the tumor. In contrast, gain-only samples exhibited scores <0 and did not differ from samples without alterations. When assessing the H3K27me2 and H3K27me3 scores separately, no difference was observed between gain-only samples and samples with no alteration. However, IHC data were only available for a small number of samples with a gain at the *EZH2* locus (*n*=7).

### Impact of *EZH2* alterations on gene transcriptional profiles

A key function of EZH2 is to repress proliferation checkpoint genes to transiently suppress GC B-cell differentiation.^14^ It has been previously demonstrated that mutant EZH2 protein aberrantly represses genes through increased promoter H3K27 tri-methylation and by exaggerating the epigenetic silencing of normal GC B-cell targets of wild-type EZH2.^14^ To gain insight into which transcriptional programs are changed in patients carrying the *EZH2* mutation, we analyzed differential gene expression patterns between mutated and non-altered patients (that is, no gain or mutation). A total of 2 276 genes were differentially expressed at a 5% FDR (1271 upregulated and 1005 downregulated genes in patients with the *EZH2* mutation compared to non-altered patients; Supplementary Table S2). Comparison with MSigDB gene sets showed that tumors with the *EZH2* mutation had enriched proliferation genes, including MYC targets, compared to wild-type samples (some of the most significant categories were HALLMARK_G2M_CHECKPOINT, HALLMARK_E2F_TARGETS, HALLMARK_MYC_TARGETS_V1, Supplementary Table S3). Enrichment analysis of our *EZH2* signature showed significant enrichment of a previously reported *EZH2* signature (*P*=2 × 10^−21^).^10^

**Figure 3:**
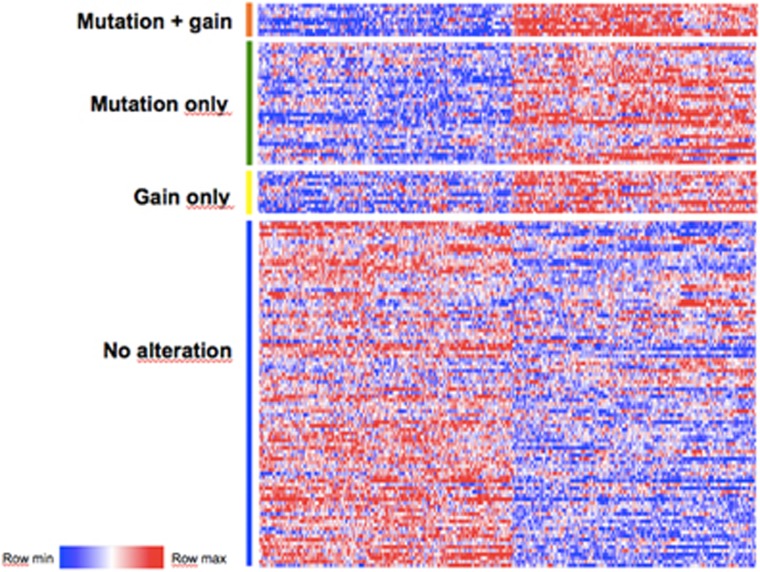
Tumors with *EZH2* gains display a similar transcriptional profile to mutated tumors. The heat map shows the 500 genes most differentially expressed between altered and non-altered tumors for all categories of tumors (gain-only, mutation-only, mutation+gain or no alteration). For a full list of differentially expressed genes in each category, refer to Supplementary Tables S2 and S4.

To examine whether gains at the *EZH2* locus would also impact the gene expression profile, we performed a differential expression gene set over-representation analysis between patients with a gain at the *EZH2* locus (gain-only) and non-altered patients. A total of 2431 genes were differentially expressed (1263 upregulated and 1168 downregulated genes in patients with an *EZH2* gain compared to non-altered patients; Supplementary Table S4). Of these, 843 genes were found in common with the previous set of genes differentially expressed in mutated samples (440 upregulated and 403 downregulated common genes in both sets), thus showing a highly significant overlap (*P*=10^−206^) between these analyses (Figure 3). Comparison with MSigDB gene sets also showed a striking similarity in categories enriched between both analyses and, to a lesser extent, an expected enrichment of genes located on chromosome 7 (Supplementary Table S5). Moreover, the enrichment of the previously reported *EZH2* signature was maintained (*P*=3.7 × 10^−5^). Overall, these results suggest that a gain at the *EZH2* locus may have similar and significant consequences at the transcriptional level to those observed in mutated tumors.

### Prognostic value of *EZH2* alterations

**Figure 4:**
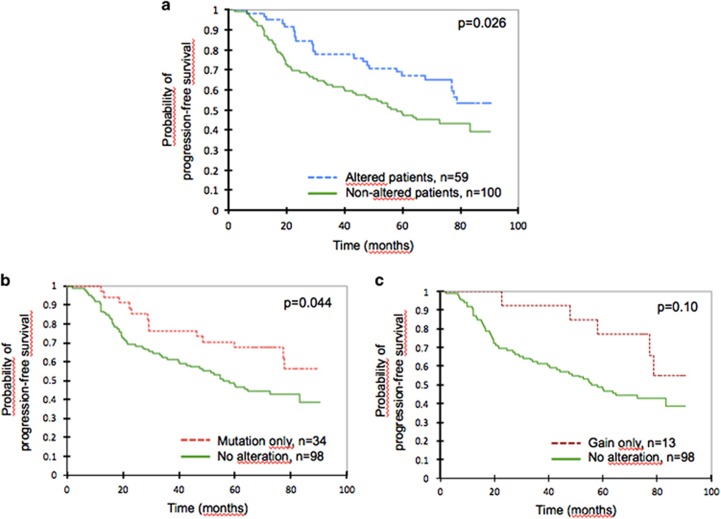
Kaplan–Meier estimates of PFS according to *EZH2* alteration type. (**a**) Any *EZH2* alteration: patients with a mutation, a gain in copy-number or both were compared to patients without alteration. (**b**) *EZH2* mutation status: patients with a sole mutation in *EZH2* (no gain detected by SNP-arrays) were compared to patients without alteration (no mutation and no gain). (**c**) Copy-number status of the *EZH2* locus: patients with a sole gain at *EZH2* locus (no mutation detected) were compared to patients without alteration (no mutation and no gain). Groups were compared using a log-rank test.

The presence of an alteration (gain and/or mutation) in *EZH2* had a favorable impact on PFS (*P*=0.026, Figure 4a). Non-altered patients had a median PFS of 58 months, whereas it was not reached for patients carrying an *EZH2* alteration. Analysis of the individual impact of mutation status or copy-number status on PFS showed the same trend, although it was limited (mutation-only versus no alteration, *P*=0.044; gain-only versus no alteration, *P*=0.10, Figures 4b and c), which may be caused by an insufficient number of patients in these subgroups needed to demonstrate a significant effect.

Early relapse within 24 months after diagnosis has recently been shown to define a group of patients at high risk of death.^31, 32^ The 2-year progression rate in our cohort was 15% (95% CI: 9–28%) in the group of patients carrying an alteration in *EZH2* and raised to 31% (95% CI: 23–41%) in non-altered patients (*P*=0.036). This favorable prognostic effect appeared to be related to both the individual impact of mutation status (15% of 2-year progression in patients with a sole mutation versus 31% for non-altered patients, *P*=0.08) or copy-number status (8% of 2-year progression in patients with a sole gain versus 31% for those with no alteration, *P*=0.11).

**Figure 5:**
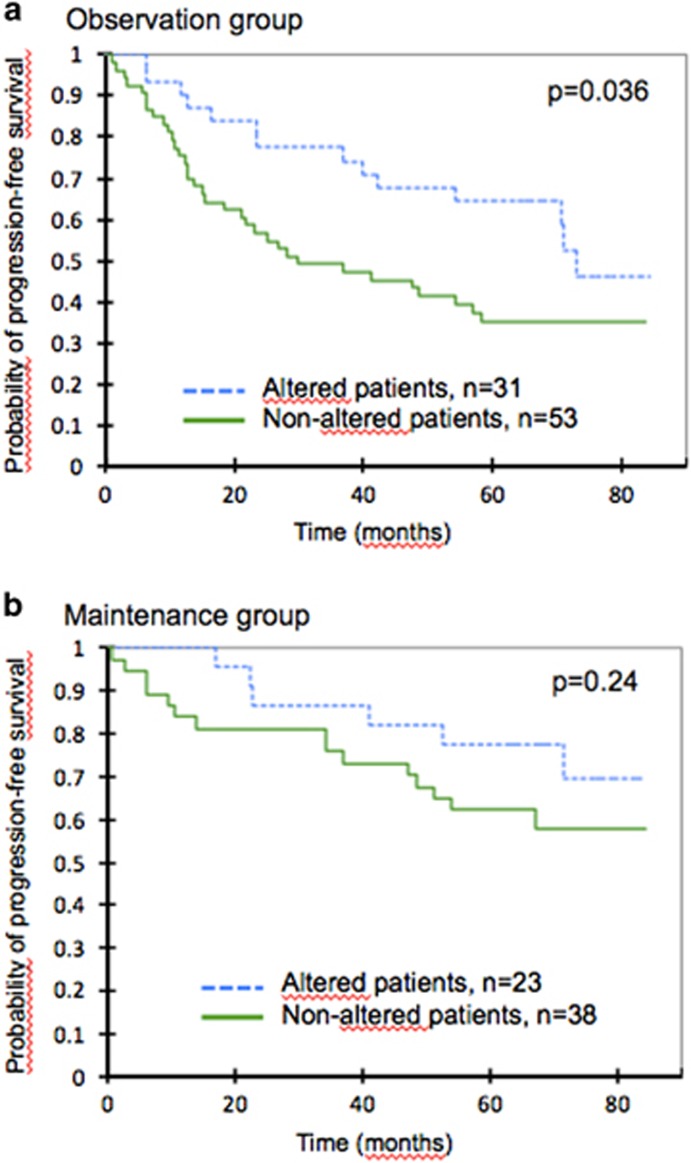
Kaplan–Meier estimates of PFS according to *EZH2* alteration (mutation and/or gain in copy-number) in the observation group (**a**) and the maintenance group (**b**). Of the 159 patients included in this study, 145 were randomized (observation group, *n*=84 and maintenance group, *n*=61). Groups were compared using a log-rank test.

When analyzing each PRIMA maintenance treatment group separately, a favorable impact of *EZH2* alterations was detected for patients in the observation group (*P*=0.036), but the significant impact was lost for patients who received the rituximab maintenance (*P*=0.24, Figure 5). No effect was detected for overall survival (data not shown). The *EZH2* mRNA expression level, the EZH2 IHC level, and the H3K27me3/me2 score could not predict the risk of progression (data not shown).

In a multivariate Cox regression model stratified on treatment group that included the *EZH2* genetic status and the FLIPI score, the presence of an *EZH2* alteration remained the only independent factor significantly associated with a better PFS (hazard ratio 0.58, 95% CI 0.36–0.93, *P*=0.025).

## Discussion

Using a comprehensive set of techniques, we have analyzed *EZH2* mutation, copy-number status and expression level in a cohort of FL patients from a prospective clinical trial. Mutations were identified in 46 of the 159 patients (29%), which is in accordance with recent reports using an NGS approach.^5, 9, 10^ A gain at the 7q36.1 locus was detected in 15% of patients and was not correlated with mutation status. Overall, 37% of patients carried an alteration in the *EZH2* gene (that is, mutation, gain or both).

We hypothesized that both gain-of-function mechanisms may participate in the deregulation of EZH2 function in FL cells. A clear impact on H3K27me2 and H3K27me3 IHC expression was observed in tumors carrying a mutation in *EZH2*, demonstrating the potential of this recently described score to predict the presence of these mutations with 100% sensitivity and specificity in FL cases. Moreover, a differential expression analysis between mutated and non-altered (no mutation and no gain) patient transcriptional profiles showed a very distinct gene expression signature related to *EZH2* mutation status, as previously reported.^5, 10^ Tumors harboring a gain at the *EZH2* locus had a significantly higher level of EZH2 protein, assessed by IHC, but did not show significant changes in the H3K27me3/me2 profile. This is in line with the known mechanism of action of the wild-type EZH2 protein, which does not show a preferential action on H3K27me2 by itself but cooperates with the mutated protein to increase H3K27me3 marks. Nevertheless, here, we describe for the first time that a genomic gain at the *EZH2* locus was associated with a particular expression profile that was highly similar to that induced by mutations and showed features of proliferation. Although *EZH2* genomic gains are part of the larger gains of chromosome 7 and we cannot exclude that other genes located on chromosome 7 might influence this transcriptomic profile, the highly significant overlap with the mutation-induced profile is appealing. As EZH2 is known to repress proliferation checkpoint genes and maintain the GC phenotype by suppressing transcriptional programs required for exiting the GC reaction and terminal differentiation,^14, 33^ it is tempting to assume that both gain-of-function mechanisms might have similar physiological effects through enhanced silencing of EZH2 targets. However, further functional work is needed to specifically determine how gains at the *EZH2* locus may participate in the deregulation of EZH2 function in FL cells. A possibility could be that a higher level of wild-type EZH2 protein might result in fine in a global methylation change at the all levels (H3K27me1, H3K27me2 and H3K27me3) without evidence for a particular increase in the H3K27me3/H3K27me2 ratio. Other targets might be investigated as well, to further explore the consequences of *EZH2* gains.

We have shown that *EZH2* alteration significantly improves PFS of patients homogeneously treated in the setting of the PRIMA trial. In particular, patients with an alteration in the *EZH2* gene experienced a significantly lower rate of early relapse. Although this seems conflicting with EZH2 ability to enhance proliferation, the proliferative capacity of a tumor does not necessarily correlates with aggressiveness or stage of the disease, as recently exemplified with the pediatric-type follicular lymphoma, an indolent type of follicular lymphoma with high proliferation index.^34, 35^ Moreover, proliferative tumors respond better to chemotherapy as a higher number of cells are in cycling and can be targeted, as all patients included in the PRIMA trial received rituximab chemotherapy as induction regimen. Recently, another group reported that *EZH2* mutations might confer a favorable prognosis in FL patients and proposed to incorporate it within a new stratification risk model.^5, 36^ Although we could only find a statistically non-significant trend for the impact of the sole genomic status on patient outcomes, we suspect that such a slight effect would be better elucidated using a larger patient cohort. Considering the biological impact of gains on the tumor transcriptional profile, our findings suggest that both types of gene alterations might be worth assessing to accurately distinguish low risk from high-risk patients. In addition, it is interesting to note that rituximab maintenance seems to reduce, if not abrogate, this prognostic impact compared to patients with no maintenance therapy, as has been previously reported for other clinical-pathogenic features.^37^ This should also be assessed in other patient cohorts to rule out a potential sampling bias.

Three EZH2 inhibitors are currently being tested in phase 1 and 2 clinical trials in patients with and without *EZH2* mutations (NCT01897571, NCT02395601 and NCT02082977); however, it is presently unknown which patients experienced a more substantial benefit from this treatment. To this end, we show that the IHC H3K27me3/me2 score fully distinguished mutated from wild-type samples, thereby confirming the applicability of this score for the prediction of *EZH2* mutation in FL patients. The use of IHC as a surrogate for *EZH2* mutation analysis has been previously validated in DLBCL,^19^ but it had not to been demonstrated in FL until now. In addition to the mutational status, we suggest that consideration of the copy-number status of *EZH2* could also help to identify patients who are more likely to benefit from this targeted therapy. Both mutation status and copy-number can be assessed using targeted NGS, and it would be interesting to use both *EZH2* locus alterations as stratification criteria to design future clinical trials of EZH2 inhibitors in FL patients.

## Figures and Tables

**Table 1:** Clinical characteristics and treatment of patients with or without an *EZH2* gene alteration

	*Alteration*	*No alteration*	P
	n=*59*	n=*100*	
	n *(%)*	n *(%)*	
*Baseline characteristics*			
Age >60 years	20 (34)	31 (31)	0.73
Male sex	28 (47)	54 (54)	0.51
Ann Arbor stage III/IV	53 (90)	92 (92)	0.77
ECOG PS ⩾1	20 (34)	31 (31)	0.73
B symptoms present	8 (14)	30 (30)	0.02
BM involvement^a^	29 (54)	69 (70)	0.009
Elevated LDH^b^	27 (53)	28 (28)	0.04
Hemoglobin level <12 g/dl	11 (19)	25 (25)	0.43
β2-microglobulin ⩾3 mg/l^c^	22 (39)	36 (37)	0.86
			
*FLIPI score*			
0–1 risk factors	9 (15)	18 (18)	
2 risk factors	23 (39)	40 (40)	0.86
3–5 risk factors	27 (46)	42 (42)	

*Histological grade*			
1–2	45 (76)	88 (88)	
3A	8 (14)	6 (6)	
FL with diffuse area	3 (5)	1 (1)	0.13
FL of undetermined grade	3 (5)	5 (5)	
			
*Induction regimen*			
R-CHOP	56 (95)	94 (94)	1
R-CVP	3 (5)	6 (6)	
			
*Maintenance regimen*			
Not randomized in PRIMA trial	5 (8)	9 (9)	1
Randomized in PRIMA trial	54 (92)	91 (91)	
Observation	31 (57)	53 (58)	1
Rituximab	23 (43)	38 (42)	

Abbreviations: ECOG PS, Eastern Cooperative Oncology Group Performance Status; LDH, lactate dehydrogenase; R-CHOP, rituximab, cyclophosphamide, doxorubicin, vincristine and prednisone; R-CVP, rituximab, cyclophosphamide, vincristine and prednisone;

‘Alteration' indicates the presence of a mutation, a gain or both, whereas ‘no alteration' refers to samples with neither mutation nor gain. The correlations between EZH2 mutation or copy-number status and the initial characteristics or treatment group were assessed using Fisher's exact test.

a BM involvement data were missing or not evaluated for one patient with EZH2 alteration and five patients without EZH2 alteration.

b LDH data were missing for one patient with EZH2 alteration.

c β2-microglobulin data were missing for three patients with EZH2 alteration and three patients without EZH2 alteration.

**Table 2:** EZH2 expression and H3K27me3/me2 score according to *EZH2* alteration status

	*Total*	*Non-altered*	*Altered (mutation or gain)*^a^	*Mutation-only*^a^	*Gain-only*^a^	*Mutation+gain*^a^
Patients, *n*	159	100	59^b^	34	13	10
IHC data usable, EZH2 score/H3K27 score	55/63	34/44	21/19	10/10	7/7	4/2
EZH2 mRNA level, median (range)	12.54 (11.51–13.36)	12.42 (11.51–13.36)	12.70 (11.95–13.70)***	12.61 (11.95–13.11)***	12.87 (11.98–13.70)**	12.92 (12.64–13.19)***
EZH2 IHC (%), median (range)	30 (5–80)	30 (5–80)	50 (20–80)***	45 (20–70)^ns^	70 (40–80)***	50 (30–80)^NT^
H3K27me2 IHC (%), median (range)	100 (2–100)	100 (30–100)	70 (2–100)***	30 (5–100)***	100 (100–100)^ns^	10 (2–50)^NT^
H3K27me3 IHC (%), median (range)	70 (1–90)	70 (1–90)	70 (50–90)^ns^	65 (50–90)^ns^	70 (50–90)^ns^	70 (50–75)^NT^
me3/me2 score, median (range)	−0.32 (−1.86; 2.74)	−0.46 (−1.86; −0.14)	(−0.87; 2.74)**	1.24 (0; 2.22)***	−0.46 (−0.87; −0.14)^ns^	1.62 (0.50; 2.74)^NT^

^NT^The differences between non-altered patients and the subgroup of patients with both mutation and gain alterations were not tested (NT) due to the small sample size.

^ns^Not significant (no trend was observed, all *P*-values were >0.1). **0.001<*P*<0.01, ***P<0.001. Expression levels between groups were compared using the Mann–Whitney test.

a All subgroups were compared to non-altered patients (no gain or mutation at the EZH2 locus). Expression levels between groups were compared using Mann–Whitney test.

b Two patients were mutated but had no SNP-array analysis performed. These patients were considered to be ‘altered' but could not be included in the ‘mutation-only' subgroup nor in the ‘mutation+gain' subgroup.

## Supplementary Material

Supplementary Figures

Supplementary Tble Legends
