# Supplementary material for: EZH2 alterations in follicular lymphoma: biological and clinical correlations
Source: Blood Cancer J. 2017 Apr 21;7(4):e555–. doi: 10.1038/bcj.2017.32 (PMC5436075; doi:10.1038/bcj.2017.32)

Supplemental Figure S1: Progression-free survival according to treatment group.

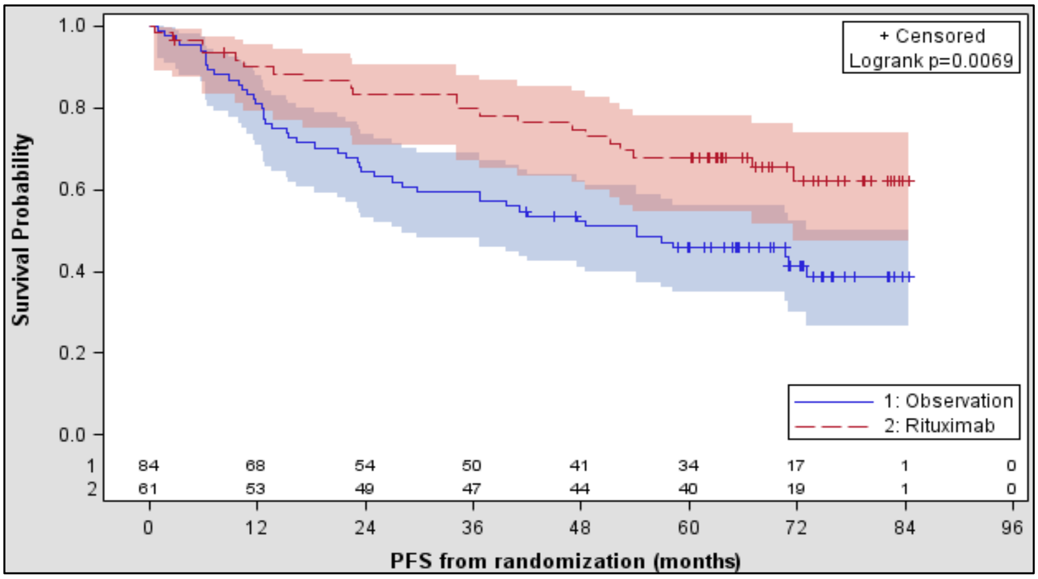

Supplemental Figure S2: Diagram showing the number of samples with available results for the different techniques applied in this study.

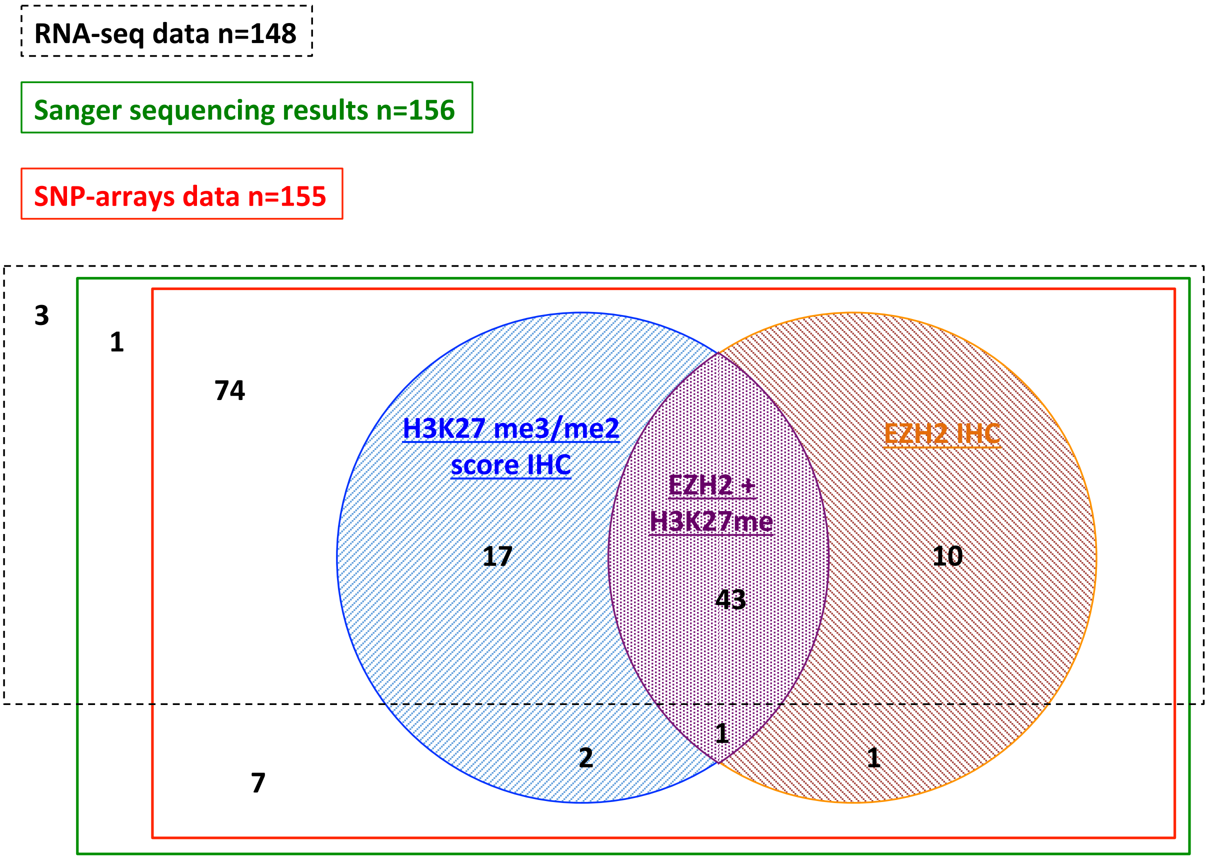

Supplement: Supplementary Figures [file bcj201732x1.pdf]
