# Supplementary material for: EZH2 alterations in follicular lymphoma: biological and clinical correlations
Source: Blood Cancer J. 2017 Apr 21;7(4):e555–. doi: 10.1038/bcj.2017.32 (PMC5436075; doi:10.1038/bcj.2017.32)
Supplement: Supplementary Tble Legends [file bcj201732x2.docx]

**Supplemental Tables**

**Supplemental Table S1: Clinical characteristics of the study cohort compared to those of the entire PRIMA population.**

The open-label, international, multicentre randomized PRIMA study enrolled a total of 1,135 patients with untreated high tumor burden FL. During the induction phase, patients were treated with one of three protocol-specified standard immunochemotherapy regimens. The three rituximab combinations used in the PRIMA study were CVP (cyclophosphamide 750 mg/m2 on day 1, vincristine 1.4 mg/m2 [capped at 2 mg] on day 1, prednisone 40 mg/m2 on days 1–5, repeated every 3 weeks for 8 cycles), CHOP (cyclophosphamide 750 mg/m2 on day 1, vincristine 1.4 mg/m2 [capped at 2 mg] on day 1, doxorubicin 50 mg/m2 on day 1, prednisone 100 mg on days 1–5, repeated every 3 weeks for 6 cycles), and FCM (fludarabine 25 mg/m2 on days 1–3, cyclophosphamide 200 mg/m2 on days 1–3, mitoxantrone 6 mg/m2 on day 1, repeated every 4 weeks for 6 cycles). Rituximab (375 mg/m2 at each infusion) was administered on day 1 of each chemotherapy course. Two additional rituximab infusions were administered in patients treated with CHOP (every 3 weeks after the last cycle) and FCM (2 weeks after the first and the fourth cycles) to ensure equivalent exposure to the antibody during induction for all patients.

Response to this induction therapy was assessed 2 to 4 weeks after the last treatment course. After this induction phase, patients who obtained a complete response (CR), an unconfirmed CR (CRu) and a partial response (PR) were randomized in a 1:1 ratio to observation or rituximab maintenance (12 infusions of 375 mg/m2 at 8 week intervals).

Patients were evaluated clinically every 8 weeks during the 2-year maintenance phase and by computed tomography scan every 6 months. Patients with bone marrow involvement at diagnosis underwent bone marrow evaluation at the end of the maintenance phase. Thereafter, a clinical evaluation and a CT scan were performed respectively every 3 and 6 months for a period of 3 years.

^1^BM involvement data were missing or not evaluated for 6 patients from the study cohort and 34 patients from the entire PRIMA population.

^2^LDH data were missing for 1 patient from the study cohort and 5 patients from the entire PRIMA population.

^3^β2-microglobulin data were missing for 6 patients from the study cohort and 85 patients from the entire PRIMA population.

^4^FLIPI score was missing for 2 patients from the entire PRIMA population.

**Supplemental Table S2: Genes differentially expressed in tumors with an EZH2 mutation compared to tumors without any *EZH2* alteration.**

**Supplemental Table S3: Enrichment of differentially regulated genes between patients with an EZH2 mutation and patients without any *EZH2* alterations.**

Most significant associations between up- and down-regulated genes in *EZH2* mutated patients and gene-sets from the MSigDB database. Only the top 50 gene-sets in each category are shown. *Abbreviations: FDR, false discovery rate; MSigDB, Molecular Signatures Database.*

**Supplemental Table S4: Genes differentially expressed in tumors with an EZH2 gain compared to tumors without any *EZH2* alteration.**

**Supplemental Table S5: Enrichment of differentially regulated genes between patients with an *EZH2* gain and patients without any *EZH2* alterations.**

Most significant associations between up- and down-regulated genes in *EZH2* patients with a gain at the *EZH2* locus and gene-sets from the MSigDB database. Only the top 50 gene-sets in each category are shown. *Abbreviations: FDR, false discovery rate.*
